# Homologous recombination deficiency status predicts response to platinum-based chemotherapy in Chinese patients with high-grade serous ovarian carcinoma

**DOI:** 10.1186/s13048-023-01129-x

**Published:** 2023-03-15

**Authors:** Zheng Feng, Di Shao, Yuhang Cai, Rui Bi, Xingzhu Ju, Dongju Chen, Chengcheng Song, Xiaojun Chen, Jin Li, Na An, Yunjin Li, Qing Zhou, Zhihui Xiu, Shida Zhu, Xiaohua Wu, Hao Wen

**Affiliations:** 1 Department of Gynecologic Oncology, Fudan University Shanghai Cancer Center, 270 DongAn Rd, Shanghai, 200032 China; 2 Department of Oncology, Shanghai Medical College, Fudan University, Shanghai, China; 3 BGI Genomics, BGI-Shenzhen, BGI Genomics, Beishan Industrial Zone, Yantian District, ShenzhenShenzhen, 518083518083 China; 4 Tianjin Medical Laboratory, BGI-Tianjin, BGI-Shenzhen, Tianjin, 300308 China; 5 Department of Pathology, Fudan University Shanghai Cancer Center, Shanghai, China

**Keywords:** Homologous Recombination Deficiency, Platinum-based Chemotherapy, High-Grade Serous Ovarian Carcinoma, Homologous Recombination Repair

## Abstract

**Background:**

Homologous Recombination Deficiency (HRD) is a predictive biomarker for ovarian cancer treated with PARP inhibitors or for breast cancer treated with first-line platinum-based chemotherapy. However, limited research is documented on platinum-based treatment prediction with HRD as a biomarker in ovarian cancer patients, especially in the Chinese population.

**Methods:**

We investigated the association between HRD status and the response of platinum-based chemotherapy in 240 Chinese HGSOC patients.

**Results:**

The Pt-sensitive patients showed higher HRD scores than Pt-resistant ones, but this was not significant(median: 42.6 vs. 31.6, p = 0.086). (Pt)-sensitive rate was higher in HRD + *BRCA*m tumors and in HRD + *BRCA*wt tumors (HRD + *BRCA*m: 97%, *p* = 0.004 and HRD + *BRCA*wt: 90%, *p* = 0.04) compared with 74% in the HRD-*BRCA*wt tumors. We also found Pt-sensitive patients tend to be enriched in patients with *BRCA* mutations or non-*BRCA* HRR pathway gene mutations (*BRCA*: 93.6% vs 75.4%, *p* < 0.001; non-*BRCA* HRR: 88.6% vs 75.4%, *p* = 0.062). Patients with HRD status positive had significantly improved PFS compared with those with HRD status negative (median PFS: 30.5 months vs. 16.8 months, Log-rank *p* = 0.001). Even for BRCAwt patients, positive HRD was also associated with better PFS than the HRD-negative group (median: 27.5 months vs 16.8 months, Log-rank *p* = 0.010). Further, we found patients with pathogenic mutations located in the DNA-binding domain (DBD) of BRCA1 had improved FPS, compared to those with mutations in other domains. (*p* = 0.03).

**Conclusions:**

The HRD status can be identified as an independent significance in Chinese HGSOC patients treated with first-line platinum-based chemotherapy.

**Supplementary Information:**

The online version contains supplementary material available at 10.1186/s13048-023-01129-x.

## Background

Epithelial ovarian cancer (EOC) is the eighth most commonly diagnosed and lethal disease among females around the world. EOC has been steadily increasing over the past 10 years in many countries, which accounts for an estimated 295,414 new cases and 184,799 deaths worldwide in 2018 [1–3]. And there are approximately 52,100 new cases and 22,500 deaths each year in China [4]. Specifically, high-grade serous ovarian carcinoma (HGSOC) is the most common subtype of EOC accounting for almost 75% of all EOCs. Unfortunately, the majority of patients are diagnosed as advanced stage III-IV at the time of preliminary diagnosis [5–7]. Historically, standard treatments for newly diagnosed EOC consisted of cytoreductive surgery and systemic platinum-based chemotherapy [8]. Recently, clinical trials have shown that maintenance therapy with Poly (ADP-ribose) polymerase (PARP) inhibitors can improve PFS, and patients with *BRCA1/2* mutation and/or homologous recombination deficiency (HRD) benefit most [9–11]. Hence, *BRCA1/2* mutation and/or HRD status served as a critical predictive biomarker for PARP inhibitors in EOC patients. Therefore, it is necessary to use a HRD test to identify the subgroup of *BRCA* wild-type patients who are likely to benefit from PARPi in the first-line maintenance setting [12].

The data from The Cancer Genome Atlas (TCGA) showed that approximately 50% of HGSOC patients have HRD [13]. HRD is caused by aberrations in genes encoding the HRR pathway, *BRCA1* or *RAD51C* promoter hypermethylation and others that may lead to genomic instability and characteristic patterns of genomic scars [13]. The classic method to determine the HRD status is the mutation detection of tumor suppressor genes *BRCA1/2* and other key genes of the HR pathway. However, the incidence of *BRCA1/2* mutations in EOCs is only approximately 30% [14, 15]. With the development of detection and sequencing technology, recent advances in the understanding of cancer genome have found that genomic scar signature will also reflect the presence of HRD [16]. If the detection of HR gene alteration is regarded as the discovery of the HRD status from the perspective of the cause, then the genomic scar analysis can determine the status of the genomic defect from the phenomenon. Genomic scar analysis as a direct and comprehensive way of predicting the HRD status of tumors had already been embedded in several clinical trials [17]. Three genomic scar signatures associated with HR deficiency derived from genome-wide copy number data have been identified as numeric sum of the loss of heterozygosity (LOH) [18], telomeric allelic imbalance (TAI) [19], and large-scale state transitions (LST) [20]. In a previous study, we developed and validated a new algorithm called genomic scar analysis (GSA) to call copy number variant and calculate HRD score by detecting above three genomic scars [21].

Up to now, there were one companion diagnostics and another complimentary diagnostic has been approved by FDA for identifying women with HRD who are likely to benefit from PARP inhibitors based on three clinical trials (NCT03737643, NCT02354586, and NCT01968213) [22–24]. Notably, one of them had optimized and verified their threshold in patients with platinum-based chemotherapy before the approval, and another had refined their threshold in two different studies [10, 25]. In September 2022, Olaparib has been approved in China for the maintenance treatment of adult patients with HRD-positive epithelial ovarian cancer who are in complete or partial response to 1st-line platinum-based chemotherapy in combination with bevacizumab. However, the HRD test kit for use as a companion diagnostic has not yet been approved in China. Therefore, there is still a lack of data on the distribution of HRD status and related research data on the correlation between HRD status and clinical features.

Given that previous studies have confirmed that both HRD scores and HRD status are correlated with the efficacy of platinum-based chemotherapy, this study aims to analyze the relationship between the HRD status of Chinese patients with ovarian cancer and the efficacy of platinum-based chemotherapy. Furtherly, this study also evaluates the proportion of HRD status in Chinese ovarian cancer patients, the correlation between HRD scores and *BRCA1/2* mutations, and its correlation with the efficacy of platinum-containing chemotherapy. This study will provide a powerful data basis for platinum-based chemotherapy efficacy prediction biomarker in Chinese ovarian cancer patient.

## Results

### Patient Characteristics

A total of 249 HGSOC patients were included in this study. The first patient in this prospective study was enrolled on January 6, 2016, and the last patient was enrolled on September 25, 2018. Nine patients with failure of quality control of sample or sequencing data or lost follow-up information were further removed for data analysis. Patient characteristics and clinical data are summarized in Table 1. The median age at diagnosis was 53 years (ranger 36–83 years). 76.7% of the patients have been diagnosed with FIGO stage III and 42 patients were FIGO IV stage (17.5%). All patients underwent surgical removal and then received platinum-based chemotherapy. One hundred and ten patients achieved R0 resection with no macroscopic disease (45.8%). Pt-sensitive patients with a platinum-free interval (PFI) of over six months accounted for 82.5 percent of the entire cohort (198/240).

**Table 1:** Clinical characteristics of HRR Cohort (*n* = 240)

	**Pt-resistant (*N* = 42)**	**Pt-sensitive (*N* = 198)**	**Total (*N* = 240)**	***p*-value**
**Age**				0.928
Median (SD)	54 (8.4)	53 (8.8)	53 (8.7)	
Range	37.0—69.0	36.0- 83.0	36.0—83.0	
**FIGO stage**				0.038
II	0 (0%)	14 (7.1%)	14 (5.8%)	
III	30 (71.4%)	154 (77.8%)	184 (76.7%)	
IV	12 (28.6%)	30 (15.2%)	42 (17.5%)	
**Residual tumor**				0.013
non-R0	30 (71.4%)	100 (50.5%)	130 (54.2%)	
R0	12 (28.6%)	98 (49.5%)	110 (45.8%)	
**CA125**				0.021
< 500 U/ml	7 (16.7%)	69 (34.8%)	76 (31.7%)	
≥ 500 U/ml	35 (83.3%)	129 (65.2%)	164 (68.3%)	
**HE4**				0.008
< 400 pmol/L	12 (28.6%)	100 (51.0%)	112 (47.1%)	
≥ 400 pmol/L	30 (71.4%)	96 (49.0%)	126 (52.9%)	
N-Miss	0	2	2	
**tBRCA**				0.003
WT	37 (88.1%)	128 (64.6%)	165 (68.8%)	
Mut	5 (11.9%)	70 (35.4%)	75 (31.2%)	
**non-BRCA HRR**				0.170
WT	35 (83.3%)	145 (73.2%)	180 (75.0%)	
Mut	7 (16.7%)	53 (26.8%)	60 (25.0%)	

Abbreviations: *tBRCA* Tumor BRCA, *HRR* Homologous recombination repair

**Fig. 1:**
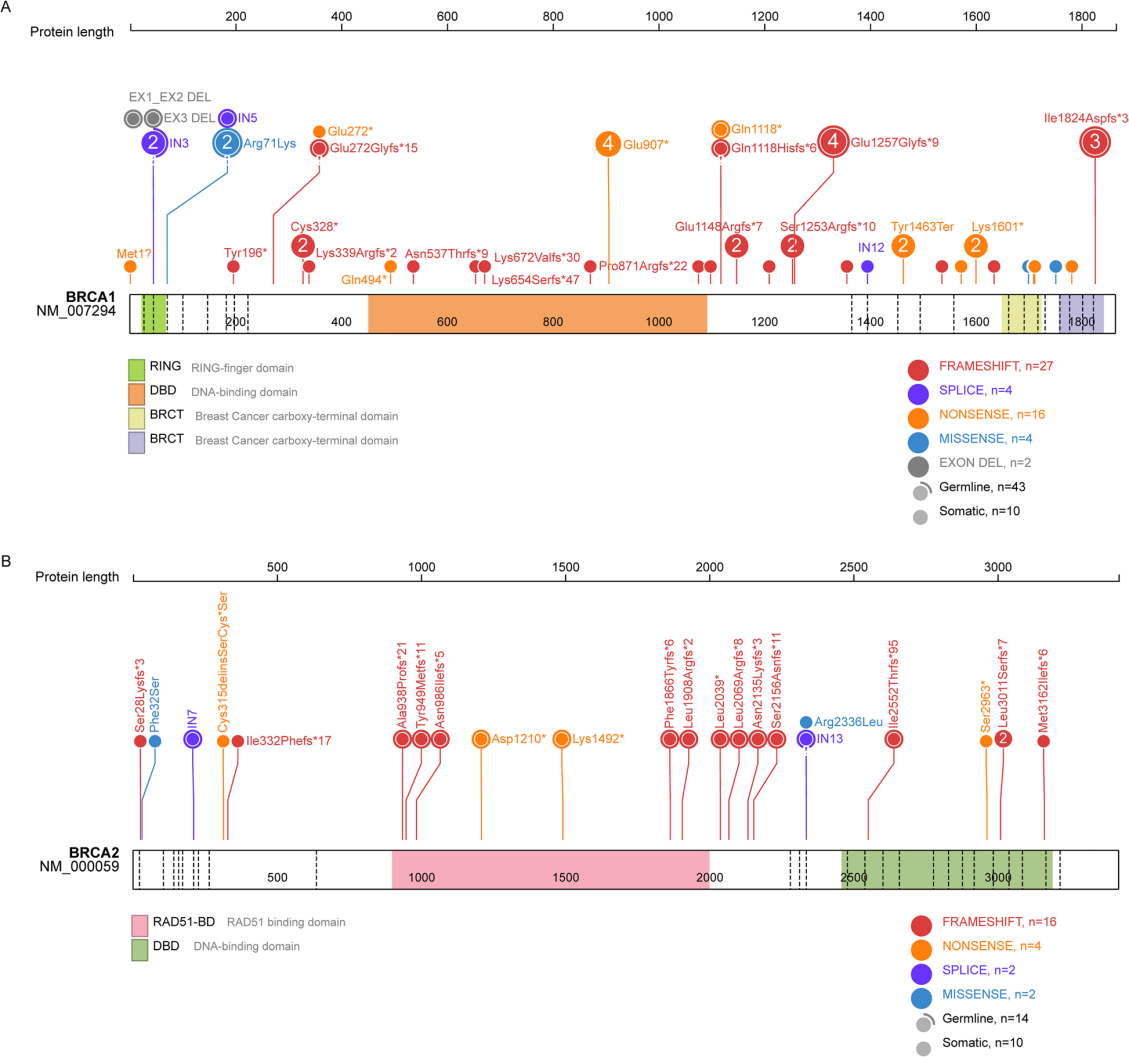
Distribution of deleterious mutations detected in *BRCA1* and *BRCA2* genes. Sticks represent mutation positions. The number represents the number of samples with the mutation (the unmarked represents 1). The colors of the bar represent the functional domains of *BRCA*

HRR gene panel test was successfully performed in all 240 HGSOC patients (HRR cohort). Average coverage alignment to the target regions was 832 (range: 645–1103) for tumor and 322 (range 282–366) for matched normal. Average percentage of reads mapped to the target region was 54.3 (range: 48.7%—58.4%)% for tumor and 57.6% (range: 50.3%-62.3%) for matched normal. Germline and somatic deleterious *BRCA1/2* mutations were observed in 31.2% of the overall HRR cohort (75 out of 240), including 53 mutations in *BRCA1*, and 22 mutations in *BRCA2* (Table 1, Fig. 1). All mutations have been previously identified in the BIC database, or are designated as deleterious, based on the nature of the mutation (nonsense, frameshift, alternate splicing, or deletion). Sixty patients (25%) had at least one deleterious mutation in a candidate HRR gene. The specific HRR mutations identified in the 14 non-*BRCA* HRR genes were: *BLM* (10, 17%), *FANCD2* (7, 12%), *RBBPB* (6, 8%), *FANCM* (5, 8%), *RAD51D* (5, 8%), *ATM* (4, 7%), *ATR* (4, 7%), *MRE11A* (4, 7%), *NBN* (4, 7%), *BRIP1* (3, 5%), *CHEK2* (3, 5%), *RAD51C* (3, 5%), *BARD1* (1, 2%), *FANCG* (1, 2%) (Supplementary Figure S1).

### HRD Score association with *BRCA1/2* and HRR mutation

**Fig. 2:**
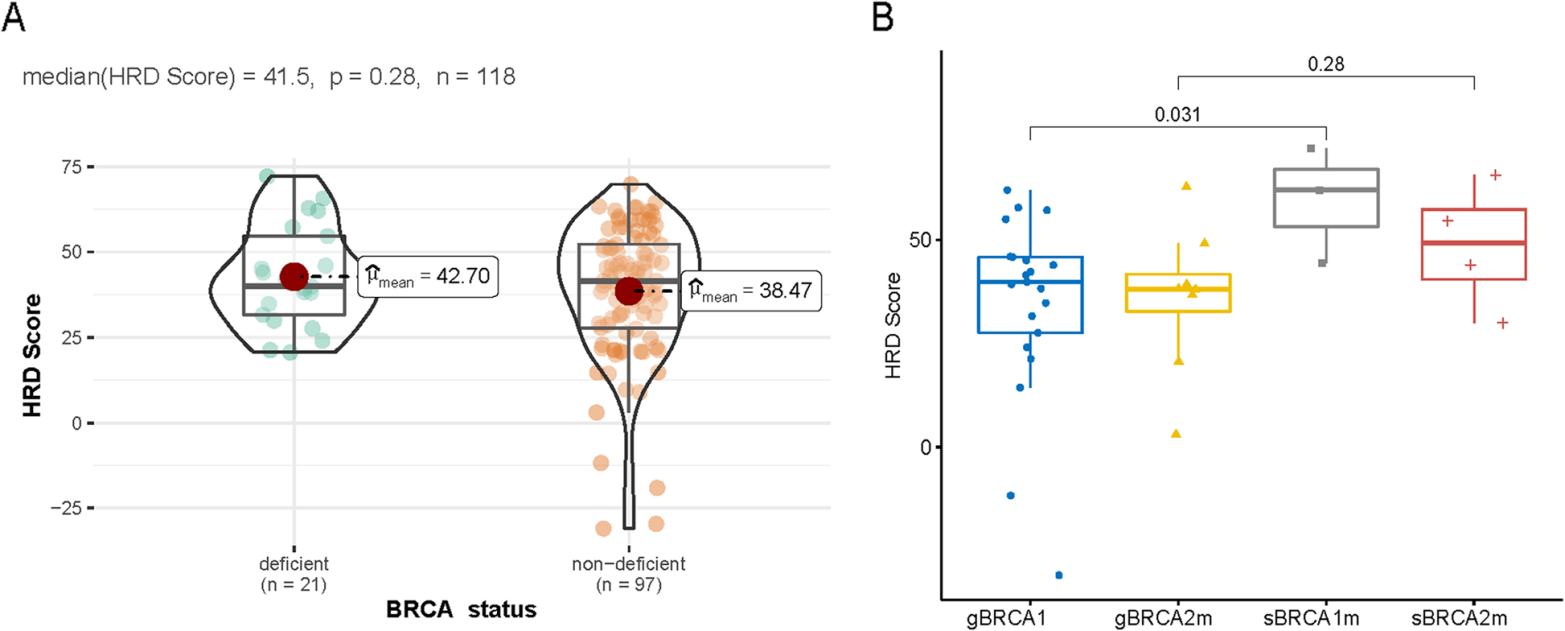
HRD score distribution in HRD cohort (*n* = 118) stratified by BRCA deficiency status (**A**) and BRCA mutation (**B**)

A subset of 118 patients from the HRR cohort had adequate DNA and underwent HRD test. All 118 patients (HRD cohort) had evaluable HRD scores, with a median of 41.5 (Fig. 2A). Of these, 17.8% (21/118) had apparent biallelic alterations in *BRCA*, based on presence of LOH or two detectable pathogenic alterations, and these cases had a mean HRD score of 42.7 compared to a mean of 38.5 for cases lacking evidence of biallelic alteration (*p* = 0.28; Fig. 2A). We also found patient with somatic pathogenicity had a higher HRD score than germline ones in *BRCA1*-deleterious patients (*BRCA1*: medium HRD score 62.1 vs 39.9, *p* = 0.031) (Fig. 2B). Subsequently, we investigated the connection between the HRD score and candidate HRR pathway gene mutations other than *BRCA* mutations. There was no significant difference in the HRD score among the HRR gene mutation groups (Supplementary Figure S2).

### HRR mutation, HRD Status, and response to platinum-based chemotherapy

**Fig. 3:**
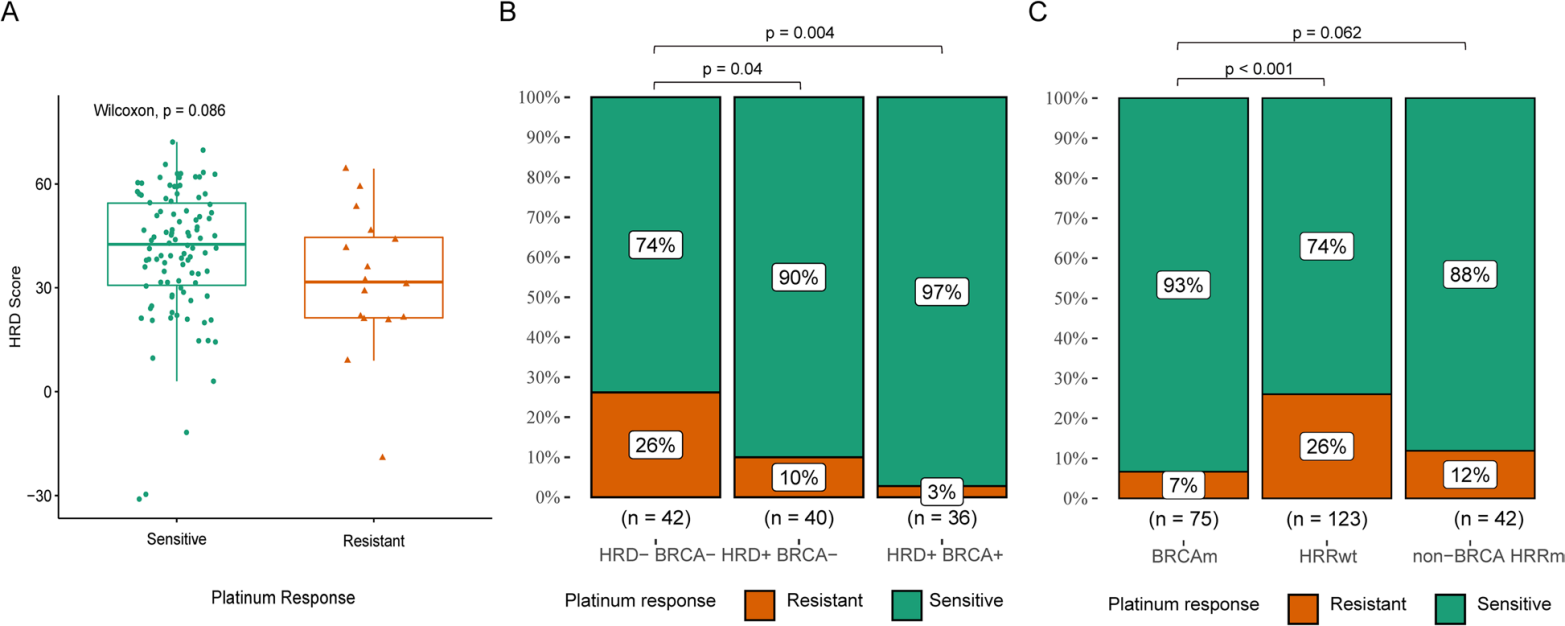
HRD score, homologous recombination mutations, and HRD status predict platinum response. The Pt-sensitive patients showed significantly higher HRD scores than Pt-resistant ones (**A**). Pt-sensitive patients tend to be enriched in patients with HRD or BRCAm (**B**) and BRCA mutations or non-BRCA HRR pathway gene mutations (**C**)

Next, we validated HRD with platinum chemotherapy efficacy in the HRD cohort. Table 1 outlines patient and tumor characteristics stratified by the platinum response. The proportions of Pt-sensitive patients in the HRD cohort were 79.7% (94 out of 118). The Pt-sensitive patients showed higher HRD scores than Pt resistant ones, but this was not significant (median: 42.6 vs. 31.6, *p* = 0.086, Fig. 3A). (Pt)-sensitive rate was higher in HRD + *BRCA*m tumors (*n* = 36) and in HRD + *BRCA*wt tumors (*n* = 40) compared with 74% in the HRD-*BRCA*wt tumors (*n* = 42) (HRD + *BRCA*m: 97%, *p* = 0.004 and HRD + *BRCA*wt: 90%, *p* = 0.04) (Fig. 3B). We also found Pt-sensitive patients tend to be enriched in patients with *BRCA* mutations (*BRCA*: 93.6% vs 75.4%, *p* < 0.001) (Fig. 3C).

### Association of BRCA1/2 mutation, HRD score, and HRD status with PFS

**Fig. 4:**
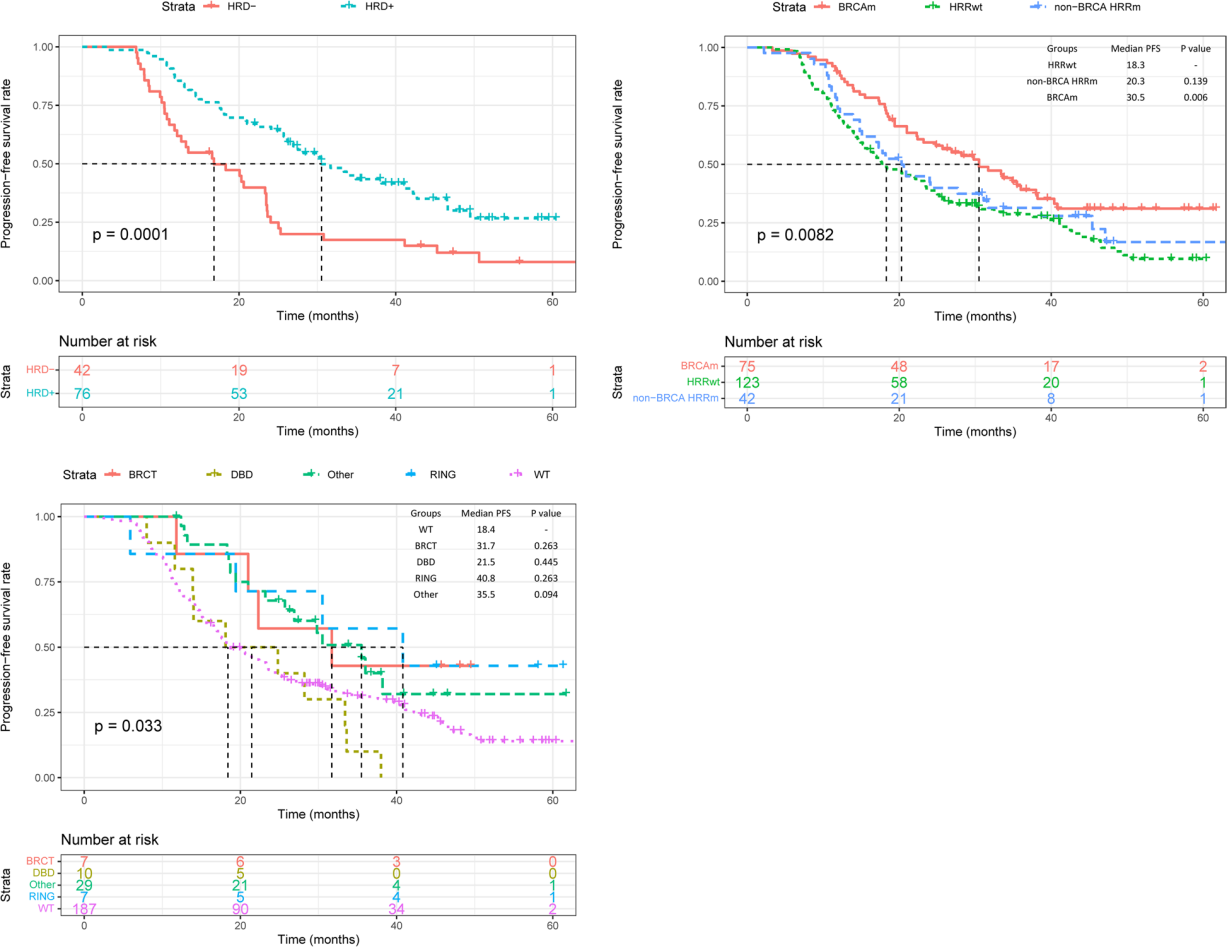
Progression-free survival by genetic status. **A**, the presence of HRD was associated with an improved PFS compared with cases without HRD. **B**, Similarly, cases with HRR gene or BRCA mutation had longer median PFS than subjects without mutation in HRR gene and BRCA. C, Patients with mutations in the DBD domain of BRCA1 are less sensitive to platinum chemotherapy

The PFS data were analyzed based on *BRCA* and HRD status in the HRD cohort. Patients with HRD status positive had significantly improved PFS compared with those HRD status was negative (median PFS: 30.5 months vs. 16.8 months, Log-rank *p* = 0.001) (Fig. 4A). Even for *BRCA*wt patients, positive HRD also associated with better PFS than the HRD-negative group (median: 27.5 months vs 16.8 months, Log-rank *p* = 0.010) (Supplementary Figure S3A).

Besides, we also evaluated whether HRR gene mutation was a prognostic factor. We found that *BRCA* mutation group had significant longer PFS than the HRRwt group (*BRCA*m: medium PFS 30.5 months vs 18.3 months, *p* = 0.006) (Fig. 4B).

A previous study suggested that mutations in the different functional domains of *BRCA* might result in differences in cancer prognosis. We defined the functional domain of *BRCA1* protein as follows: 1) the N-terminal Really Interesting New Gene (RING) domain: AA 8–96; 2) DNA-binding domain: AA 452–1092; and 3) the BRCA1 C-terminal (BRCT) domain: AA 1646–1736 and 1760–1855. Similarly, functional domains of BRCA2 were defined as 1) RAD51-binding domain (RAD51-BD): AA 900–2000; 2) DBD: AA 2459–3190. Considering these domains, 37 patients of the *BRCA* mutation group were divided into subgroups depending on the position of *BRCA* mutations, and their survival outcomes were compared. Patients with pathogenic mutations located in the DBD domain of *BRCA1* had improved FPS, compared to those with mutations in other domains. (*p* = 0.03). Due to the small sample size of some domains, this conclusion needs more research to verify.

Ten covariates (HRD status, residual tumor, t*BRCA*, HRD score, CA125, HE4, cancer history, HRR, FIGO stage, and Age) were evaluated in the univariable Cox proportional hazards regression model. The univariate analysis identified 4 covariates (HRD status: HR, 0.44; 95% CI [0.29–0.68]; *p* < 0.001; residual tumor: HR, 0.45; 95% CI [0.29–0.70]; *p* < 0.001; t*BRCA*: HR, 0.54; 95% CI [0.33–0.90]; *p* = 0 0.017; HRD score: HR, 0.64; 95% CI [0.41–0.98]; *p* = 0 0.04) as potential candidates for the multivariate model at the 0.05 alpha level based on the Wald chi-square statistic (Table 2). On multivariate analysis, residual tumor was again to be significant factors for PFS (HR, 0.47; 95% CI [0.30–0.74]; *p* = 0.001) (Table 2). We found patients with HRD-positive tumors tended to undergo R0 resection at tumor reductive surgery (Pearson's Chi-squared test, *p* = 0.03). Thus, having an HRD-positive tumor had a longer median PFS compared withthose who did not undergo R0 resection and were HRD negative whether R0 is achieved or not.(nonR0 & HRD + vs non-R0 & HRD-, *P* < 0.0083; R0 & HRD + vs non-R0 & HRD-, *P* < 0.001) (Supplementary Figure S3B).

**Table 2:** Univariable and multivariable analysis of progression-free survival in HRD cohort (*n* = 118)

Factors	Univariable Analysis			Multivariable Analysis		
	**HR**	**95%CI**	***P*** value^**a**^	**HR**	**95%CI**	***P* value**
HRD score	0.64	0.41–0.98	0.04	0.92	0.38–2.23	0.854
HRD status	0.44	0.29–0.68	< 0.001	0.62	0.22–1.71	0.356
Residual tumor	0.45	0.29–0.70	< 0.001	0.47	0.30–0.74	0.001
tBRCA	0.54	0.33–0.9	0.017	0.66	0.32–1.38	0.272

Abbreviations: *tBRCA*   Tumor BRCA, *HR*   Hazard ratio

^a^ Adjusted with a Bonferroni correction

## Discussion

Women with EOC have a higher chance to benefit from platinum-based chemotherapy and PARP inhibitor therapy if their tumor has a germline or somatic *BRCA1/2* pathogenic variant [9–11]. The American Society of Clinical Oncology (ASCO) published guidelines for germline and somatic testing in epithelial OCs [26]. The guideline recommends all women diagnosed with epithelial ovarian cancer should be offered genetic testing for *BRCA1*, *BRCA2*, and other ovarian cancer susceptibility genes, irrespective of their clinical features or family cancer history, and somatic tumor testing for *BRCA1* and *BRCA2* pathogenic or likely pathogenic variants should be performed in women who do not carry a germline pathogenic or likely pathogenic *BRCA1/2* variant. According the guidelines, at least 76.3% of patients in this study would receive two tests because they are negative for a germline variant and would need a subsequent tumor test to identify somatic *BRCA1/2* variants. In the current study, we used liquid phase hybridization and NGS which allow rapid and accurate detection of both hereditary and somatic *BRCA* and other HRR gene variants in paired blood and tumor tissue samples. In our cohort, 23.7% of tumors carry germline *BRCA1/2* disease-causing variants and approximately 7.5% of tumors have a somatic (acquired) disease-causing variant. Our clinical practice shows this universal *BRCA1/2* testing gives quick and reliable information to allow doctors to make decisions about treatment and genetic counseling. The reported frequency of *BRCA1/2* deleterious variants in patients with EOC varies between 5 and 30% and is affected by the population studied [27, 28]. In our current study, tumor *BRCA1/2* deleterious variants were identified in 31.2% of HGOCs, one quarter of these mutations are somatic. This rate is in line with previously reported rates of 18%–24% in the Chinese population.

Improved prognosis with higher partial response (PR) and complete response (CR) rates to platinum-based chemotherapy and longer PFI, has been observed in patients who are *BRCA1/2*-mutant carriers with ovarian cancer [29]. This “BRCAness” phenotype is likely due to defects in the homologous repair which might confer enhanced sensitivity to DNA crosslinking agents and PARP inhibitors [30]. Tumors that display properties of ‘BRCAness may also respond to similar therapeutic approaches. Germline or somatic mutations in HRR genes are candidates for displaying BRCAness. Pennington and colleagues demonstrated that deficiency in other homologous recombination proteins also confers sensitivity to platinum and improved OS with platinum treatment (*p* = 0.0006) [31]. In the current study, of 123 carcinomas without germline or somatic homologous recombination mutations, only 91 (73.9%) were Pt-sensitive. While, of carcinomas with a homologous recombination mutation in 14 key non-*BRCA* HRR genes, 37 (88.1%) were Pt-sensitive (*p* = 0.062). Although the difference was not but not statistically significant, a trend of longer PFS was observed in patients with a germline or somatic HRR mutation (medium PFS: 20.3 months vs 18.3 months, *p* = 0.139). This suggests that a wider range of HGSOC patients may benefit from the use of platinum-based chemotherapy other than solely *BRCA1* or *BRCA2* mutated patients.

For the overall cohort, the proportion of HRD score ≥ 42 is 48.3%, and the positive rate of HRD status is 64.4%, which is higher than the published studies (approximately 50%). Perhaps, this may be due to the fact this study involved only HGSOC patients, while previously reported studies included other histological subtypes [24, 32–34]. The ARIEL2 trial evaluated Rucaparib in 180 patients with Pt-sensitive recurrence EOC (97% HGSOC among all cohorts), they demonstrated that the positive rate of HRD status (HRD single signature-LOH score or *BRCA* mutation) was high was 78.69% [35]. In addition, the higher HRD positive rate was also attributed to the population studied. According to several existing studies, the incidence of HRD in high-grade serous ovarian cancer in the Chinese population is 65–68%, which is slightly higher than that in NOVA and PRIMA [36, 37]. At last, the HRD positive rate is also highly related to the method of setting the threshold. In the absence of clinical efficacy and clinical prognosis data, the HRD score threshold is mostly established based on the consistency with *BRCA1/2* deficiency, then combined with the clinical efficacy data of PARP inhibitors or platinum salts to adjust the threshold. For example, under the same HRD score detection method, the cutoff of Veliparib for advanced HGSOC is ≥ 33 [23], while ≥ 42 for Olaparib and Niraparib [24, 34]. Therefore, at the beginning of the development of detection methods and algorithms, *BRCA1/2* deficient samples can be used to establish the biological threshold, but it still needs to be combined with the clinical data of PARP inhibitors or platinum salts to verify or adjust the threshold. We demonstrated that positive HRD could predict higher platinum sensitivity and better clinical outcomes, even in *BRCA*wt patients. It seems that HRD tests, beyond *BRCA* mutant, are most likely to identify subgroups of HGSCs that derive different magnitudes of benefit from platinum-based chemotherapy and PARP inhibitor.

Previous studies have shown *BRCA1/2* mutations could predict sensitivity to platinum-based chemotherapy in triple-negative breast cancer (TNBC) tumors and ovarian cancer tumors [38, 39]. To identify more patients who could benefit from platinum chemotherapy, we hypothesized that HRD-positive HGSOC patients would show improved sensitive to platinum chemotherapy than HRD-negative and thus have better clinical outcomes. To date, only a few abstracts have investigated the association of HRD status and platinum-based chemotherapy in epithelial ovarian cancer [40, 41]. In this study, we found HRD-positive patients had a higher (Pt)-sensitive rate than HRD-negative patients regardless of *BRCA* mutation status. We also demonstrated patients who had HRD-positive tumors also had a better PFS when compared to the patients with HRD-negative tumors. Univariate analysis also shown significant association with both PFS and HRD status. This result is consistent with previously published observations in ovarian cancer and supports the hypothesis that HRD status could predicts sensitivity to platinum chemotherapy.

## Methods

### Patients

Tumor collection for this study was approved by by the Institutional Reviewer Board of Fudan University Shanghai Cancer Center and BGI (NO. 1703170–15 and NO. BGI-IRB 19,151-T2).Informed written consent was obtained from all individual patients. Eligible patients were aged 18 years or older and had stage II-IV HGSOC confirmed by pathological examination. Patients entering the study were required to have received two or more previous courses of platinum-based chemotherapy. Clinical data including age, family history, preoperative laboratory data, pathological diagnosis, tumor FIGO stage, surgical outcomes, patients’ disease status were obtained from medical records. Surgical outcomes were categorized as R0 and non-R0 regarding the residual disease. Patients who recurred in six months or after the last platinum treatment are labeled as Pt-sensitive, while those who recurred in < 6 months from the last platinum are considered as Pt-resistant. The response was evaluated according to RECIST version 1.1, and PFS was defined as the time from surgery until objective tumor progression or death.

### Extraction of DNA from tumor and paired blood samples

The formalin fixation and paraffin embedding (FFPE) tissue samples and paired blood were obtained from 240 ovarian cancer patients who had undergone surgery at the Fudan University Shanghai Cancer Center, clinical characteristics are shown in Table 1. Genomic DNA (gDNA) was extracted from FFPE tissue sections from each available tumor sample by QIAamp DNA FFPE TISSUE KIT (Qiagen), according to the manufacturer’s instructions. Besides, genomic DNA was extracted from paired blood using QIAamp DNA Blood Midi Kit (Qiagen), according to the manufacturer’s instructions. Qubit fluorometer 3.0 (Invitrogen) was used for DNA quantification, and 1% Agarose Gel Electrophoresis was used to determine DNA quality. Extracted gDNA was sheared into fragments, then the library was constructed by CoBox adaptors, which is a patented design by BGI Genomics Co., Ltd. with UMI (Unique Molecular Identifier) and dual Index, which can effectively reduce background noise and make variation detected correctly.

### Targeted hybridization capture and sequencing

Genome-wide SNPs data were generated using a custom hybridization enrichment panel (Roche, Basel, Switzerland), which targets 93,200 SNPs distributed across the human genome, called the HRD panel below. All coding exons and intron–exon boundaries (± 20 base pairs) of homologous recombination repair (HRR) were enriched by a custom capture chip (BGI Genomics, Shenzhen) which included hereditary risk-related gene and DNA repair pathway genes relevant to gynecological oncology. Enriched DNA samples were sequenced by 100-bp pair-end reads performed using the MGISEQ-2000 platform (MGI Tech Co., Ltd.). The average sequencing depth of tissue samples should exceed 150 × for the HRD panel, and the average sequencing depth of tissue and blood samples needs at least 500X for *BRCA1/2* and other HRR genes. HRR mutation positive was defined as pathogenic and likely pathogenic mutations in the following 24 HRR pathway genes as *ATM*, *BRCA1*, *BRCA2*, *ATR*, *BARD1*, *BLM*, *BRIP1*, *CHEK2*, *MRE11A*, *NBN*, *PALB2*, *RAD51C*, *RAD51D*, *RBBP8*, *SLX4*, *XRCC2*, *FANCA*, *FANCC*, *FANCD2*, *FANCM*, *FANCG*, *FANCL*. These genes predicted to impact HRR pathway when mutated was selected based on review of the available literature.

### Detection of HRD score and mutations

HRD score was calculated by a genomic scar analysis algorithm ASGAD (Allele-Specific Gene-scar Analysis tool for Diagnosis) [41]. ASGAD can be used to measure the LOH, TAI, and the LST in gDNA isolated from FFPE tumor tissue specimens. Meanwhile, the differences in purity and ploidy of tumor tissue were also taken into account in the algorithm. The raw sequence data were filtered and mapped to the human genome (hg19) using BWA aligner 0.7.17. Local alignment optimization, variant calling and annotation were performed using GATK toolkit 3.2, and VarScan. Variants with population frequency over 0.1% in the ExAC, 1000 Genomes, and dbSNP databases were excluded from further analysis. The remaining variants were annotated using VEP software and interpreted following the “Genetic Variation Annotation Standards and Guidelines” (2015 Edition) issued by the American College of Medical Genetics (ACMG) for germline mutation, and the “Cancer mutation interpretation of guidelines and standards (2017 Edition)” for somatic mutation, respectively. Gene variants were named according to HGVS (Human Genome Variation Society; http://www.hgvs.org/).

### HRD status assessments

The assessment of HR deficiency status requires combining the HRD score and *tumor BRCA1/2* mutations status. Tumor *BRCA1/2* positive is defined as pathogenic or likely pathogenic mutation, otherwise, it will be defined as tumor *BRCA1/2* negative. HRD score ≥ 42 was defined as high HRD score and the optimal *P*-value and the highest statistic were achieved when using Kaplan–Meier analyses with Log-rank Test in predicting PFS (Supplementary Figure S4). Tumors are considered as HRD status positive if the HRD score is high (above the biological HRD score threshold, ≥ 42) or tumor *BRCA1/2* positive. The tumors are HRD status negative if the HRD score is low (below the score threshold, < 42) and tumor *BRCA1/2* negative. HR deficiency status could not be analyzed comprehensively if either HRD score analysis or the tumor *BRCA1/2* mutation was detected failed.

### Survival analysis

Multivariable (adjusted for FIGO stage and residual tumor) Cox proportional hazards (PH) models were used for multivariate survival analyses, and *P* values were based on the Likelihood ratio test. The hazard ratios (HR) and 95% confidence intervals were also reported. Categorical variables, including t*BRCA* mutation status, HRD score, HRD score in the *BRCA1/2* wildtype, and HRD status, were also evaluated with Kaplan–Meier (KM) curves, and *P* values were based on Log-rank tests.

### Statistical analysis

All statistical analysis was conducted using R version 3.6.1 (R Core Team, 2013) with an α of 0.05. The statistical tools employed in this study include the Student's t-test and one-way ANOVA analysis of variance. All reported *P* values were two-sided. *P* < 0.05 was considered to be statistically significant.

## Supplementary Information

**Additional file 1:** **Figure S1.** Determine the Optimal cut point for HRD score based on the log-Rank statistic.

**Additional file 2: Figure S2.** Frequency of HRR gene mutation in 240 HGSOC patients.

**Additional file 3: Figure S3.** HRD score by HRR gene mutation in HRD cohort (*n* = 118).

**Additional file 4: Figure S4.** Progression-free survival based on R0 resection and HRD status.

## Data Availability

The data that support the findings of this study have been deposited into CNGB Sequence Archive (CNSA) of China National GeneBank DataBase (CNGBdb) with accession number CNP0001456.

## Abbreviations

ACMG: American College of Medical Genetics
FFPE: Formalin fixation and paraffin embedding
ASCO: American Society of Clinical Oncology
RAD51-BD: RAD51-binding domain
BRCT: BRCA1 C-terminal
RING: Really Interesting New Gene
LOH: Loss of heterozygosity
TAI: Telomeric allelic imbalance
LST: Large-scale state transitions
PARP: Poly(ADP-ribose) polymerase
HGSOC: High-grade serous ovarian carcinoma
TCGA: The Cancer Genome Atlas
EOC: Epithelial ovarian cancer
