# Supplementary figures and images for: Homologous recombination deficiency status predicts response to platinum-based chemotherapy in Chinese patients with high-grade serous ovarian carcinoma
Source: J Ovarian Res. 2023 Mar 15;16:53. doi: 10.1186/s13048-023-01129-x (PMC10015784; doi:10.1186/s13048-023-01129-x)

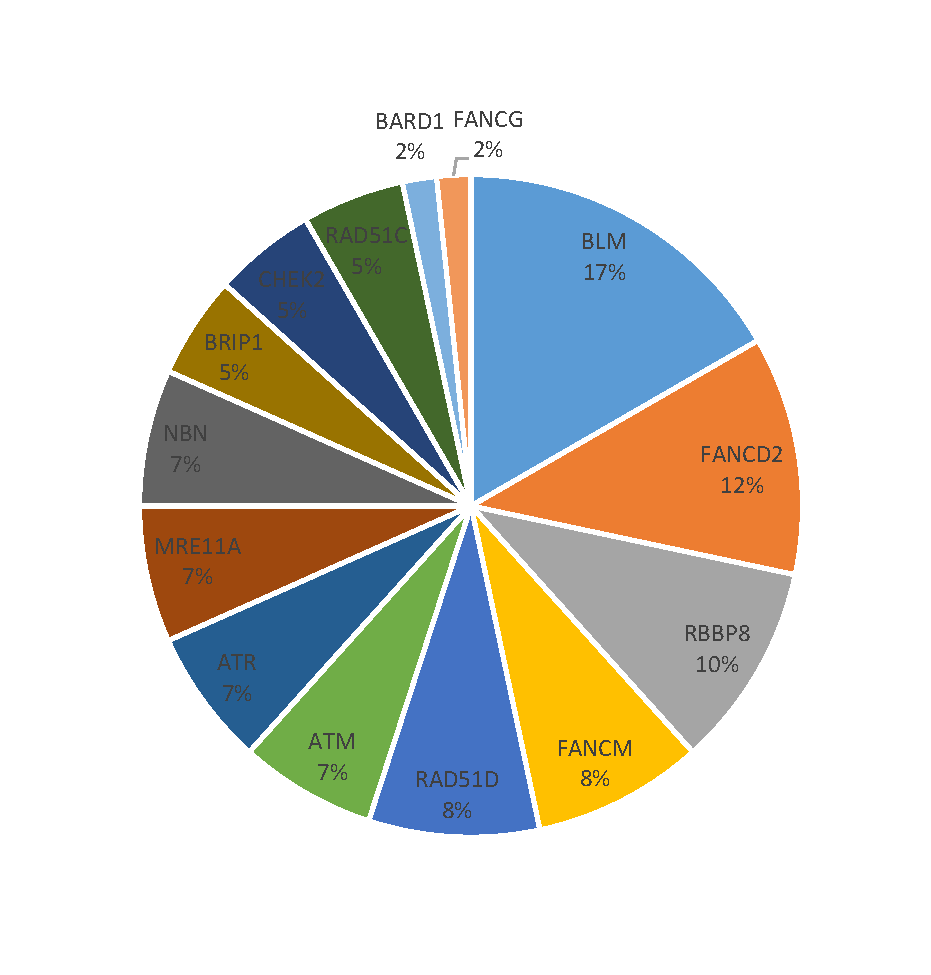

Supplement: Supplementary file 1 — Additional file 1: Figure S1. Determine the Optimal cut point for HRD score based on the log-Rank statistic. [file 13048_2023_1129_MOESM1_ESM.tif]

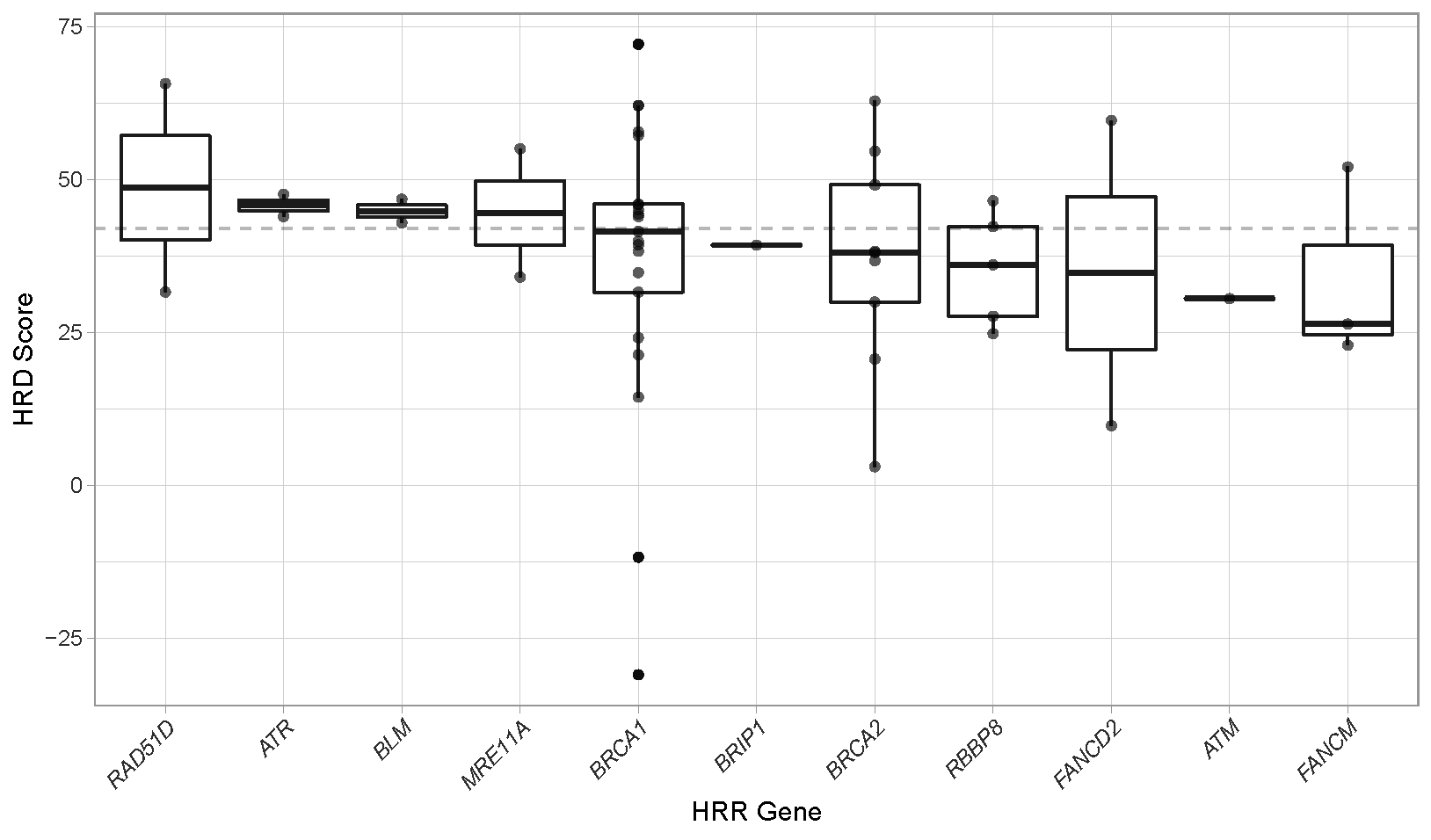

Supplement: Supplementary file 2 — Additional file 2: Figure S2. Frequency of HRR gene mutation in 240 HGSOC patients. [file 13048_2023_1129_MOESM2_ESM.tif]

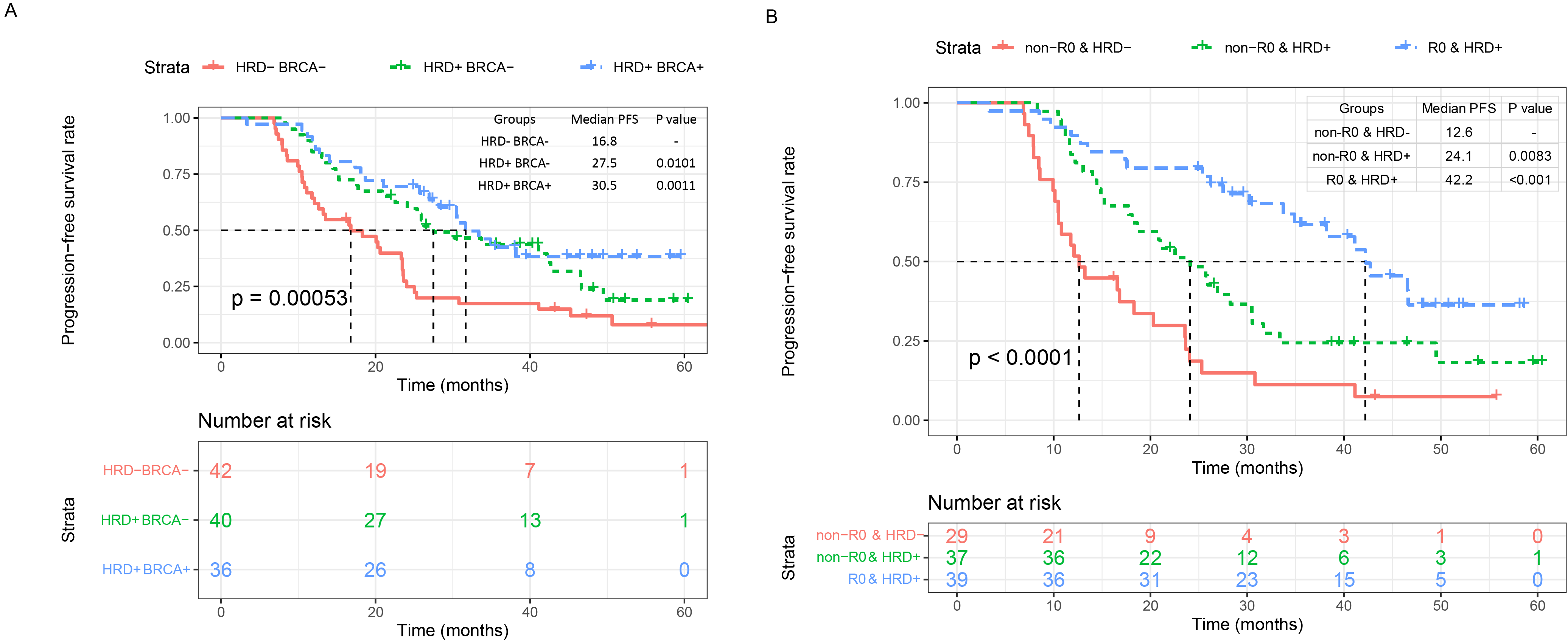

Supplement: Supplementary file 3 — Additional file 3: Figure S3. HRD score by HRR gene mutation in HRD cohort (n = 118). [file 13048_2023_1129_MOESM3_ESM.tif]

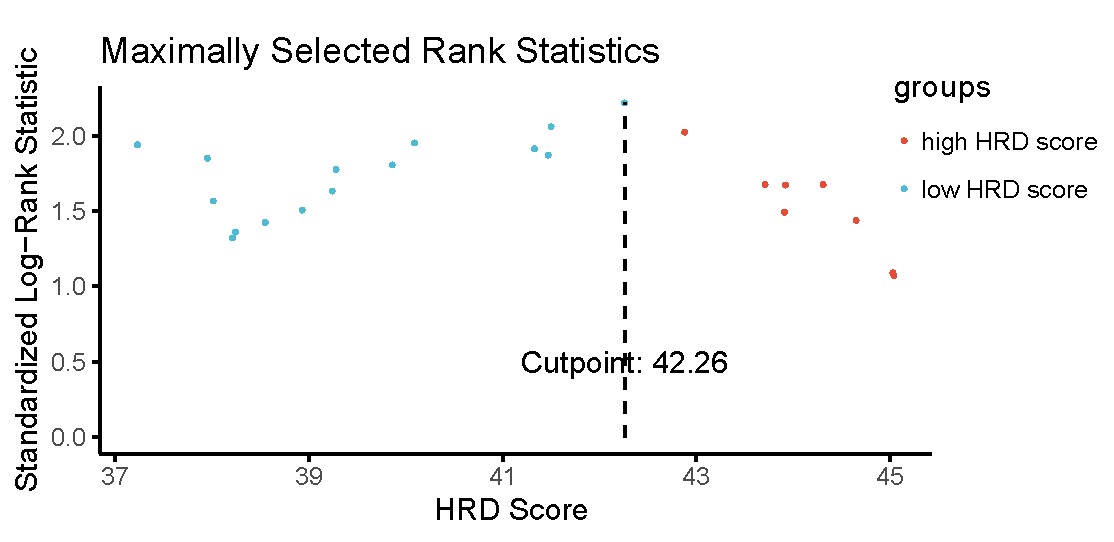

Supplement: Supplementary file 4 — Additional file 4: Figure S4. Progression-free survival based on R0 resection and HRD status. [file 13048_2023_1129_MOESM4_ESM.tif]
